# Barriers to Clinical Research in Latin America

**DOI:** 10.3389/fpubh.2017.00057

**Published:** 2017-04-18

**Authors:** Kathryn Chomsky-Higgins, Theodore A. Miclau, Madeline C. Mackechnie, Dino Aguilar, Jorge Rubio Avila, Fernando Baldy dos Reis, Roberto Balmaseda, Antonio Barquet, Alfredo Ceballos, Fernando Contreras, Igor Escalante, Nelson Elias, Sergio Iriarte Vincenti, Christian Lozano, Fryda Medina, Gavino Merchan, Julio Segovia, Enrique Guerado, Jose Eduardo Quintero, Saam Morshed, Mohit Bhandari, Theodore Miclau

**Affiliations:** ^1^San Francisco – East Bay Surgery Program, University of California, Oakland, CA, USA; ^2^Stanford University, Palo Alto, CA, USA; ^3^Department of Orthopaedic Surgery, Institute for Global Orthopaedics and Traumatology (IGOT), University of California, San Francisco, CA, USA; ^4^Department of Orthopaedic Surgery, Orthopaedic Trauma Institute, Zuckerberg San Francisco General Hospital and Trauma Center, San Francisco, CA, USA; ^5^Hospital Vivian Pellas, Managua, Nicaragua; ^6^Clinica Medica Sur, Guadalajara, Mexico; ^7^Federal University of Sao Paulo, Sao Paulo, Brazil; ^8^Department of Orthopaedic Surgery, Centro de Investigaciones Médico-Quirúricas (CIMEQ), La Habana, Cuba; ^9^Department of Traumatology and Orthopaedics, AEPSM, Montevideo, Uruguay; ^10^Department of Orthopaedic Surgery, Centro de Investigaciones Médico-Quirúricas (CIMEQ), La Habana, Cuba; ^11^Hospital San Juan de Dios, San José, Costa Rica; ^12^Hospital Universitario de Caracas, Caracas, Venezuela; ^13^Vila Velha Hospital, Espírito-Santo, Brazil; ^14^Hospital San Gabriel and Clinica del Sur, La Paz, Bolivia; ^15^Clinica Anglo Americana, Lima, Peru; ^16^Hospital de Traumtologia Instituto Mexicano del Seguro Social, Mexico City, Mexico; ^17^Hospital de la Policia Nacional, Guayquil, Ecuador; ^18^Instituto de Prevision Social, Servicio de Ortopaedia y Traumatologia, Asunción, Paraguay; ^19^Department of Orthopaedic Surgery, Traumatology, and Rehabilitation, University of Malaga, Hospital Costa del Sol, Marbella, Málaga, Spain; ^20^Hospital Universitario San Jorge, Clinica de Ortopedia y Traumatología, Pereira-Risaralda, Colombia; ^21^Division of Orthopaedic Surgery, McMaster University, Hamilton, ON, Canada

**Keywords:** barriers, clinical research, capacity, sustainability, Latin America

## Abstract

Enhancing health research capacity in developing countries is a global health priority. Understanding the orthopedic burden of disease in Latin America will require close partnership between more-developed and less-developed countries. To this end, the Osteosynthesis and Trauma Care Foundation assembled a research consortium of Latin-American orthopedic leaders. Prior to the meeting, we surveyed attendees on perceived barriers to conducting research at their institutions. During the event, working groups discussed these barriers, developed strategies for addressing them, and planned future steps for collaboration. The participants established the need for global relationships that allow colleagues from Latin America to access to training and established investigational infrastructure of North American centers to address research questions relevant to their communities. As a result of the discussion, the International Orthopaedic Multicenter Study (INORMUS) in Fracture Care was initiated. Since then, an expanded international working group, Associación de Cirujanos Traumatológicos en las Americas (ACTUAR), has been created with the purpose of promoting increased global partnership for research capacity development.

## Introduction

Musculoskeletal conditions contribute to an increasing burden of disease throughout the world, including Latin America. The most financially and socioeconomically impactful of these conditions is musculoskeletal trauma, which is on the rise as a direct result of road traffic accidents. The World Health Organization predicts that road traffic injuries will increase from the ninth to the third highest cause of Disability-Adjusted Life Years (DALYs) worldwide by 2030 (1). According to data from the 2013 Global Burden of Disease study, injuries have already become the fifth leading cause of DALYs (2). As this burden continues to rise, estimates of orthopedic injury patterns and prevalent treatment strategies in low and middle income countries (LMICs) become ever more important for health systems planning. Developing these estimates will require collaboration between more-developed (Global North) and less-developed (Global South) countries, as many in the latter group lack robust research infrastructure (3, 4). This results in decreased research productivity; accordingly, articles originating from Latin-American countries are underrepresented in major orthopedic surgery journals (5, 6). This is a fundamental limitation to the development of knowledge that could improve musculoskeletal injury care and inform research and policy priorities.

Many authors recognize the potential of sustainable, collaborative North–South partnerships to improve clinical research and education among LMICs (5–14). Despite broad recognition of this issue for over two decades and the current support for institutional partnerships as a solution, there is still a persistent lack of research infrastructure in developing countries. Thus, the Council on Health Research for Development (COHRED) organized a series of meetings on Latin-American health research priorities in which representatives from 20 Latin-American countries agreed to implement national and regional health research programs (15, 16). While many recommendations have resulted from these high-level discussions, little information exists regarding specific barriers that could help to inform ground-level North–South partnerships in the near future. These authors could find no literature on the barriers to orthopedic research in Latin America. To understand these specific barriers and develop the consortium, we assembled a working group of leaders from 13 Latin-American countries in a professional forum to discuss barriers faced in clinical research and to develop strategies for surmounting these obstacles.

## Methods

Prior to the Osteosynthesis and Trauma Care Foundation Forum of the Americas meeting in October 2011, we sent a needs assessment document to stimulate discussion. Attendees were asked to query their colleagues in suggested areas to achieve representation from their regions. Participants from this expert working group included 15 participants representing 13 different countries: Argentina, Bolivia, Brazil, Chile, Colombia, Costa Rica, Ecuador, Mexico, Nicaragua, Paraguay, Peru, Uruguay, and Venezuela. Additional participants from Japan, China, the Netherlands, and the United States were also invited to provide contrasting perspectives. The course Chairmen selected the surgeons based on their interests in international orthopedic activities and commitment to improving the field of orthopedics through research.

The areas discussed included their current role and extent of research at each participant’s home institution, and their personal involvement in research activities. The meeting was conducted in English, as all of the participants also were proficient in that language. Pre-meeting questions were circulated to stimulate discussion prior to the meeting, and comments were distributed and used in working group sessions. During these sessions, group members discussed barriers to research at their hospitals, possible strategies for overcoming these barriers, and research questions that were priorities for their patients. A scribe recorded the proceedings for each group. Finally, each group presented their findings to the entire assembly and discussed plans for collaborative research. The focus of this report is to review those barriers faced specifically in Latin America.

## Results

Current research programs vary from non-existent to fairly robust. Approximately two-thirds of the participants reported that their institutions participate in research of some kind. Most also noted that research was a requirement for professional advancement, either for graduation or matriculation in a program or for promotion or advancement within their institutions. Approximately half reported current involvement in research activities. About one-third were interested in research but not currently involved in a project, and a few noted that residents were the ones that exclusively completed research at his or her hospital. Where research was performed, most noted that the work included general topics (often initiated by residents’ research questions), while some had specific areas of interest and expertise.

There was complete agreement that research capacity and interest could increase if appropriate financial and structural support were in place. Participants reported that clinical research support staff as well as financial support would likely improve the research capacity at their home institutions. Approximately half affirmed that access to scientific journals would also be a supportive measure. Other suggestions included free-of-cost trial implants, increased support from other colleagues, development of a research mentality, and establishment of a clinical database. Participants indicated that financial incentives would be a principle stimulator of interest in clinical research among colleagues at their institutions. This recognition might also come in the form of scholarships to present findings at national and international conferences. Increased access to hardware, support staff, and protected time could also be motivators. According to this group of orthopedists, research interest would increase if there were opportunities to work collectively in multidisciplinary groups or with other research centers. Some suggested that the opportunity to answer clinical questions of relevance to their patients would be motivation enough to stimulate research activity.

**Figure 1:**
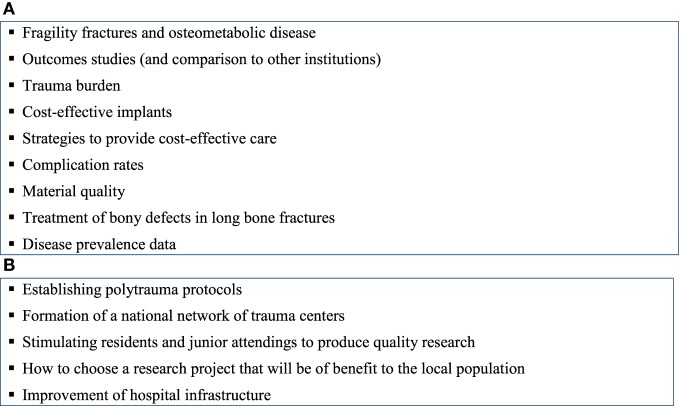
**(A)** Key clinical questions identified by working group. **(B)** Key tasks identified by working group for development of research infrastructure.

Participants identified several key clinical questions and tasks for their practice settings (Figures 1A,B). Topics included outcomes, cost-effectiveness, trauma burden, specific treatment queries, and quality of materials. Tasks included hospital infrastructure development, establishment of polytrauma protocols, formation of a network of trauma centers, and clinical research design. Nearly all respondents indicated interest in a clinical research symposium on the fundamentals of clinical research, and all indicated interest in receiving email updates on topics in evidence-based medicine. All affirmed that they would be interested in participating in an international multicenter research project on orthopedic trauma.

The working groups each produced a list of anticipated or known barriers to conducting research in their settings. These lists were reviewed individually by the larger group. It was clear from these discussions that barriers exist along all steps in the research process, from unfamiliarity with evidence-based medicine techniques to publication bias. In parallel, the working groups recommended strategies for surmounting these barriers and identified tasks for initiating the process of overcoming them (Table 1).

**Table 1:** **Barriers to research paired with potential interventions for overcoming them**.

Barrier	Tasks to overcome barrier
Lack of evidence-based medicine knowledge	Find models of EBM at conferences, in journals
	Target residents (who may have to complete research projects for advancement in their programs)
	Participate in collaborative study with a small, manageable question
Lack of journal access and/or lack of ability to read journals in English	Attempt to get access to journal articles *via* World Health Organization, preferably in Spanish
Insufficient institutional interest	Target departments chiefs to achieve buy-in
Insufficient number of patients	Consider multicenter trial
Use of residents as research staff is problematic due to turnover	Consider short-term study
Lack of incentives and academic recognition makes it difficult to get physicians involved	Seek support specifically for motivating incentives or scholarships
Problematic charting makes retrospective studies difficult	Consider prospective study
Lack of funding and access to grants	Partner with University that has access to funding
Publication bias (against researchers from developing countries); local journals not particularly active	Partner with University that has access to journals
Political issues	–
Lack of support for ethics approval	Join another study with appropriate ethics approval
As patients must pay for their own implants, randomization in implant studies is not possible	Consider alternative study designs and seek donated plates
Patient follow-up is very difficult	Consider what information can be gained in a single visit
Lack of start-up funds and critical personnel	Choose a simple research question that will not require vast expenditures

## Discussion

The international research summit provoked thought on the real challenges that potential Latin-American researchers might face in performing clinical investigations in their hospitals. The groups produced a list of solutions for overcoming specifically identified barriers. The participants confirmed the need for global North–South relationships that allow South colleagues access to training and established investigational infrastructure of the North to address research questions relevant to their communities. More importantly, the process allowed for discussion around the diverse experiences of each professional and galvanized commitment to persevere through these challenges. Though considerable published work exists on the imperative to build such partnerships, there is little writing on the methodology for doing so, particularly among surgeons.

Those present at the meeting made a commitment to collaborate on International Orthopaedic Multicenter Study (INORMUS) in Latin America. This project aligns with recommendations from the 2008 COHRED Conference for Latin-American Research and Innovation for Health which advocated increased epidemiological study of the burden of disease in Latin America in order to inform resource allocation (16). INORMUS is a short-term fracture audit that may serve as a bridge to a longer-term trauma registry. Each site will have access to the data from all sites, thus providing a database with adequate power to answer clinical questions that Latin-American orthopedists may face in their settings. We anticipate that the process of working together on this project will engender strong working relationships across institutions that the data from INORMUS will fill an important gap in burden of disease knowledge, and that implementation of the project will provide a framework for novel research infrastructure in Global South settings.

With the enthusiasm and success of INORMUS, orthopedic surgeons involved in this project have sought to create an expanded international collaborative initiative focused on building research capacity across institutions throughout Latin America. The product of this pioneering endeavor is Associación de Cirujanos Traumatológicos en las Americas (ACTUAR), a consortium established to support investigative and networking opportunities across the Americas. Though only recently established, ACTUAR has already had promising responses from orthopedists representing over 15 different countries across the Americas. The inaugural meeting of these surgeons will be held in the near future in order to discuss how this initiative can best address the barriers to conducting research in Latin America. ACTUAR aims to increase research capacity through sustainable, collaborative partnerships and to ultimately improve fracture care and to guide policy priorities.

## Author Contributions

All authors participated in the discussion and intellectual contribution that was the foundation of the article. The draft manuscript was written by KC-H, TAM, MM, and TM. Each of the authors has critically reviewed the content, approved the final version to be published, and agreed to be accountable for all aspects of the work.

## Conflict of Interest Statement

The authors declare that the research was conducted in the absence of any commercial or financial relationships that could be construed as a potential conflict of interest.
